# A Policy and Environmental Response to Overweight in Childhood: The Impact of Gold Medal Schools

**Published:** 2008-09-15

**Authors:** Brad L Neiger, Rosemary Thackeray, Carl L Hanson, Jonathan W Anderson, Sarah Rigby, Chelsea Hussey

**Affiliations:** Department of Health Science; Brigham Young University, Provo, Utah; Brigham Young University, Provo, Utah; Brigham Young University, Provo, Utah; Utah Department of Health, Salt Lake City, Utah; Utah Department of Health, Salt Lake City, Utah

## Abstract

**Background:**

The prevalence of overweight among US children and adolescents has increased substantially since 1980. As a result, overweight in childhood and adolescence has become a substantial health problem that requires effective health promotion programs and interventions.

**Context:**

Coinciding with the 2002 Winter Olympic Games in Salt Lake City, the Utah Department of Health (UDOH) developed a pilot program called Gold Medal Schools (GMS) to promote healthy lifestyles among school-aged children.

**Methods:**

The GMS program was designed to help schools develop policies and create healthy school environments to meet specific criteria at 5 levels: bronze, silver, gold, platinum, and platinum focus. Participating schools, mentored by the UDOH, earn incentives to create a healthy school environment.

**Consequences:**

A total of 316 schools and approximately 166,600 students in 37 Utah school districts have participated in the GMS program. As a result, 1,029 medals have been awarded, 2,205 policies have been developed, and 2,121 environmental changes have been reported since program inception (2001-2002 school year).

**Interpretation:**

Because of their participation in the GMS program, schools have developed and implemented a wide range of school-based policies and environmental changes. To improve the program, we recommend enhanced efforts in impact and outcome evaluation and increased participation in vigorous-intensity physical activity during the school day.

## Background

The prevalence of overweight (body mass index [BMI] at or above the 95th percentile) among US children aged 6 to 11 years increased from 6.5% to 18.8% from 1980 through 2004 (1). During the same time, overweight more than tripled among adolescents aged 12 to 19 years, increasing from 5% to 17.4% (1). When including children who are at risk for overweight (BMI at or above the 85th percentile), these estimates double (2). By comparing 3 sets of National Health and Nutrition Examination Survey data for 1999-2004, we estimated that one-third of children (aged 6-11) and adolescents (aged 12-19) are either overweight or are at risk for becoming overweight (3). These data lead to the commonly held belief that overweight is the most important health problem facing children in the United States (2).

A basic concern of overweight in childhood is that it may lead to obesity in adulthood. In a comprehensive longitudinal study predicting obesity in adulthood from childhood and parental obesity, the probability of obesity in adulthood exceeded 50% among overweight children aged 6 years or older compared with approximately 10% for nonoverweight children (4). The same study found that 79% of those aged 10 to 14 years who were overweight in childhood and had at least 1 obese parent were obese as adults (4).

Although overweight in childhood and adolescence increases the risk of obesity and many chronic diseases in adulthood, the burden of overweight manifests during childhood and adolescence. One of the earliest and most direct problems associated with overweight as perceived by children themselves is social discrimination, which in turn is associated with poor self-esteem and depression (5). The social impact of overweight in childhood and adolescence may have lasting effects on self-esteem, body image, and economic mobility (6). As with obesity among adults, the burden of overweight during childhood involves orthopedic, neurologic, pulmonary, gastroenterologic, and endocrine conditions (6). Overweight in childhood or adolescence is associated with hypertension, dyslipidemia, and type 2 diabetes (7,8).

Designing and implementing effective programs and interventions that promote health among children and adolescents and that decrease overweight and related social and biological burdens should be a priority for health promotion and disease prevention. Programs that address the primary risk factors related to overweight within policy and environmental frameworks are important (9).

## Context

In 1995, Salt Lake City, Utah, was selected as the host city for the 2002 Winter Olympic Games. In conjunction with the Olympics, a health committee was organized by the Utah Department of Health (UDOH), the Utah Medical Association, and the Utah Public Health Association in June 1999 and was named A Healthier You 2002 to encourage and promote healthy lifestyles in Utah. In 2001, staff from the UDOH developed a pilot program called Gold Medal Schools (GMS) that was later incorporated into A Healthier You 2002.

UDOH designed the GMS program for use in elementary schools. Its primary mission was to develop or improve policies and environments to support healthy eating, physical activity, and a tobacco-free lifestyle. The program was largely influenced by the Utah State Office of Education's core curriculum (curriculum standards by subject for each grade level) and guidelines from the Centers for Disease Control and Prevention to address overweight and obesity in elementary schools. Today, the GMS program represents a collaborative effort involving several organizations including the UDOH, Utah's 12 local health departments, the Utah State Office of Education, the Utah PTA, and the Utah affiliates of the American Heart Association and the American Diabetes Association. In April 2005, the UDOH and Intermountain Healthcare announced a 5-year partnership wherein Intermountain Healthcare granted $1.5 million to expand the GMS program. The program is also funded, in part, by the Preventive Health and Health Services Block Grant and the George S. and Dolores Doré Eccles Foundation.

## Methods

Any public, private, or charter elementary school in Utah may apply to become a GMS. The program is designed to help schools develop, promote, and enforce policies and create healthy school environments to meet specific criteria at 5 levels: 1) bronze, 2) silver, 3) gold, 4) platinum, and 5) platinum focus. A school reaches platinum focus level when it effectively implements all criteria for GMS and meets goals for a comprehensive school health program. Schools can implement bronze through gold level requirements during a 1- to 3-year period. However, the platinum level requires exclusive implementation during 1 full year, and the platinum focus areas must be completed exclusively 1 year at a time.

To achieve the bronze medal award, a school must complete 6 criteria: 1) write a policy requiring 90 minutes (45 minutes for kindergarten) of structured physical activity each week, using the Utah State Office of Education's physical education core curriculum; 2) teach the health education core curriculum provided by the Utah State Office of Education; 3) establish a Gold Medal Mile walking program; 4) write a policy that promotes safe routes to school by requiring the development and distribution of a routing plan for child access to schools; 5) write a policy mandating a tobacco-free school; and 6) complete and submit the Utah School Heart Health Survey. This survey, used to collect baseline data on school policies and practices related to the program, is completed by teachers, a school administrator, and the food service coordinator.

To achieve the silver medal award, a school must continue implementing the bronze criteria and complete 3 silver criteria: 1) the parent-teacher organization (PTA or PTO) must coordinate at least 1 health event per year that involves students, parents, teachers, and the principal; 2) write a policy for faculty and staff wellness activities and ensure faculty awareness of these activities; and 3) offer various competitive and noncompetitive physical activity programs accessible to all students. To achieve the silver medal award, the school must also complete 3 activities from an additional list of criteria that includes 17 activities (eg, walk your child to school day, 5-A-Day grocery store tours, life skills training).

To achieve the gold medal award, a school must continue implementing the bronze and silver criteria and complete 4 gold criteria: 1) write a policy that requires all physical education and physical activity courses to be overseen by certified physical education teachers or physical education specialists employed by the school or school district; 2) write a policy for all teachers and staff to ensure food is not used as a reward or punishment for students and ensure faculty awareness of and compliance with the policy; 3) complete 2 activities beyond the silver medal level from the additional criteria list; and 4) complete 4 criteria from the *Change the Scene* program. The *Change the Scene* program allows schools to choose from 9 different nutrition policy-related activities, including writing a policy requiring heart-healthy food choices outside school meal services (eg, classroom parties, PTA fundraisers), offering nutrition education in the classroom and dining room, and writing a policy requiring recess to be scheduled immediately before lunch.

To achieve the platinum medal award, a school must continue implementing the bronze, silver, and gold criteria and complete 5 platinum criteria: 1) require the school community council (a state-mandated school organization composed of parents or guardians, PTA representatives, administrators, and school employees) to include health topics on the agenda at each of its meetings; 2) write a policy requiring healthy choices for all foods and beverages at school events, in vending machines, and in other school venues; 3) create a year-long faculty/staff wellness program; 4) involve families and the community in completing GMS criteria; and 5) write a policy that requires recess to be scheduled immediately before lunch, or write a policy that requires adequate time for eating lunch. Lunch must be 20-30 minutes in duration and occur between 11:00 AM and 1:00 PM.

To achieve the platinum focus award, a school must continue implementing the bronze, silver, gold, and platinum criteria and develop 1 project each year from a list of 9 focus areas until the school has addressed all areas. A project from the mental health and wellness focus area must be completed first. Schools then select projects from the remaining options: asthma, diabetes control, environmental quality, fruits and vegetables, immunizations, injury prevention, oral health, and sun safety. Each project has a menu of options, though 1 option is typically a required component (eg, with respect to asthma, the required component is developing a system to track students with asthma). Then the school chooses 2 additional components from a list of 4 items (eg, asthma training for faculty and staff, using the Winning with Asthma training program [10] for faculty and staff who direct physical education and extracurricular activities).

Before the beginning of each academic year, the GMS program sends a recruitment packet to each elementary school that is not currently participating in the program. Personnel from local health departments also visit these schools and usually meet with the principal to provide information about the program. To have a successful GMS program, support is required from 3 key participants: a mentor, the principal, and the school coordinator. The UDOH provides a mentor for each school to assist in the implementation of the GMS program. The mentor, who may serve up to 6 schools, has a clear understanding of the program, guides and supports schools to complete all criteria, attends all training meetings, visits schools once each week, and completes baseline assessments with new schools. Mentors also provide several support services that vary by school and school district. Mentors may be involved in activities such as helping coordinate kick-off and awards assemblies, developing a bulletin board, writing newsletter articles, helping coordinate the staff and faculty wellness program, participating with students in walking programs, assisting with record keeping as well as implementation of the Gold Medal Mile, and developing policies and writing reports. The principal champions the GMS program, gains support from faculty and staff, reviews and signs policies, and then helps promote and enforce those policies. The school coordinator is a school employee who volunteers his or her time, works closely with the mentor, and is responsible for internal communication and coordination. The mentor and school coordinator often share or divide responsibilities listed as mentor responsibilities. In addition to these 3 participants, the corresponding local health department provides support, as does the Heart Disease and Stroke Prevention Program at the UDOH, involving staff experts in nutrition and physical activity.

Participating schools may earn up to $1,500 in awards ($200 for bronze, $300 for silver, $500 for gold, $300 for platinum, and $200 for the mental-health platinum focus). Schools can use cash awards for new physical education equipment, nutrition resources, or materials for preventing tobacco use. Some schools have purchased salad bar stations, and others have used their awards to build a walking track around the school. As schools commit to participate in the GMS program, they are also provided several resources, including training sessions; a $100 stipend each year the school is active in the program; $50 in tobacco-use prevention incentives; *Energizers*, a booklet to assist schools with the requirement to provide 90 minutes of physical education (11); and *Rewards Kids Will Crave*, a booklet related to nonfood incentives (12).

## Consequences

To date, 316 schools and approximately 166,600 students from 37 school districts have participated in the program. These data correspond to 2,009 individuals receiving GMS program training since the program inception (2001-2002 school year). As of the 2007-2008 school year, 284 schools in 34 of Utah's 40 school districts are active in the program.

A total of 2,205 policies and 2,121 environmental changes have been developed. Participating schools submit midyear progress reports and year-end reports that describe both progress toward and completion of policies and environmental changes. UDOH reviews the information and enters it into a database. School mentors work with their assigned schools to ensure that each policy developed is also implemented. For example, GMS program data collected in the 2006-2007 school year revealed that 87% of participating schools implemented their worksite health promotion policy. Development and implementation of policies and environmental changes correspond to 1,029 medals awarded since the 2001-2002 school year: 300 bronze, 270 silver, 222 gold, 130 platinum, and 107 platinum focus (reaching at least the mental health and wellness focus area). As a result, UDOH distributed $296,600 in cash awards or incentives from academic years 2001-2002 through 2007-2008.

Each year the GMS program collects and reports process evaluation measures. However, impact or outcome data have not been systematically collected. Data from 1 study related to changes in BMI percentiles, physical activity, and dietary habits have been collected but not yet published. Certain data from the Utah School Heart Health Survey also relate to impact measures. For example, data collected from teachers indicate that students involved in the GMS program receive 90 minutes of physical education per week substantially more often than students in schools that do not participate in the program. Teachers involved with the GMS program also use food as a reward substantially less often than do teachers from schools not participating in the program.

## Interpretation

Schools have long been viewed as the optimal setting for promoting health among children. The Surgeon General's 2001 *Call to Action to Prevent and Decrease Overweight and Obesity* (13) and the 2005 Institute of Medicine's (IOM) report *Preventing Childhood Obesity: Health in the Balance* (14) each include specific action steps for school boards, administrators, staff, and students to improve physical activity and nutritional status and to reduce childhood obesity. A substantial proportion of these recommendations relate to school-based polices, food quality, and educational instruction. The GMS program has incorporated nearly all of these action steps into the GMS criteria.

The 2 areas in which the GMS program should be improved to meet IOM recommendations are 1) ensuring that all children and youth participate in a minimum of 30 minutes of moderate- to vigorous-intensity physical activity during the school day and 2) implementing annual assessments of each student's height, weight, and sex- and age-specific BMI percentile, and making this information available to parents or guardians. The current GMS guideline is 90 minutes of structured physical activity each week, and no annual assessment of student height and weight is made.

In 2007, the IOM issued a report on the progress of preventing childhood obesity and included 4 recommendations to improve school-based prevention efforts (15). The first recommendation relates to providing resources and support for school-based policies and programs. The GMS program is meeting this standard. The other 3 recommendations pertain to evaluating school-based programs. According to the IOM report, many school-based programs are incorporating policy and programs, yet comprehensive evaluation is lacking. The GMS program does have a basic evaluation framework. The program conducts process evaluation by measuring program participation, award recognitions, and the number of policies developed. However, the program lacks specific data about the impact on student health status. The 2007 IOM report specifically identifies the need to assess the link between changes in school environment and health or behavioral outcomes. The GMS program could enhance its efforts in this area. A recent effort in Arkansas demonstrates that student assessment can be done (16). In 2003, the state passed Act 1220 (17) that requires Arkansas public schools to annually measure each child's BMI and report the results to parents or guardians. The student participation rate has ranged from 94% to 97%, and researchers estimate that this act has helped curb the increase in childhood obesity rates in Arkansas.

The GMS program has achieved good participation rates, and, as a result, schools are implementing many policy and environmental changes recommended by the Surgeon General, the IOM, and various other researchers and entities. By enhancing evaluation efforts, the GMS program can contribute to the evidence base of successful programs and in time be considered a model program for other schools and health departments in other states.
